# Kumquat Fruit Administration Counteracts Dysmetabolism-Related Neurodegeneration and the Associated Brain Insulin Resistance in the High-Fat Diet-Fed Mice

**DOI:** 10.3390/ijms26073077

**Published:** 2025-03-27

**Authors:** Alessandro Massaro, Pasquale Calvi, Ignazio Restivo, Marta Giardina, Flavia Mulè, Luisa Tesoriere, Antonella Amato, Domenico Nuzzo, Pasquale Picone, Simona Terzo, Mario Allegra

**Affiliations:** 1 Dipartimento di Scienze e Tecnologie Biologiche, Chimiche e Farmaceutiche, Università degli Studi di Palermo, Viale delle Scienze, 90128 Palermo, Italy; alessandro.massaro01@unipa.it (A.M.); pasquale.calvi@unipa.it (P.C.); ignazio.restivo@unipa.it (I.R.); marta.giardina@unipa.it (M.G.); flavia.mule@unipa.it (F.M.); luisa.tesoriere@unipa.it (L.T.); antonella.amato@unipa.it (A.A.); mario.allegra@unipa.it (M.A.); 2 Institute for Biomedical Research and Innovation—IRIB, 90146 Palermo, Italy; domenico.nuzzo@irib.cnr.it (D.N.); pasquale.picone@irib.cnr.it (P.P.)

**Keywords:** Kumquat fruit, HFD, insulin resistance, neuroinflammation, neurodegeneration

## Abstract

Metabolic disorders and brain insulin resistance (IR) are major risk factors for the development of neurodegenerative conditions. Kumquat fruit (KF) administration has demonstrated significant anti-dysmetabolic effects, improving peripheral IR in murine models of metabolic syndrome. Along these lines, this study evaluated the neuroprotective effects of KF supplementation in a model of dysmetabolism-induced neuronal damage and its ability to counteract the disruption of brain insulin signalling. To this end, biochemical and histological analysis assessed neuroapoptosis, disruption of brain insulin signalling and neuroinflammation in a model of high-fat diet (HFD)-induced neuronal damage. Our findings demonstrate, for the first time, that KF supplementation significantly counteracts HFD-induced neuroapoptosis downregulating pro-apoptotic genes (*FAS*-L, *BIM* and *P27*) and upregulating the anti-apoptotic ones (*BDNF* and *BCL-2*). Coherently, KF positively influenced the expression of selected genes related to Alzheimer’s Disease. Relevantly, these effects were associated to KF ability to restore brain insulin signalling by increasing insulin receptor expression, reducing IRS-1 serine phosphorylation, enhancing both AKT activation and GSK-3β inactivation. Accordingly, KF suppressed HFD-neuroinflammation, counteracting the overexpression of NF-κB and its downstream enzymatic products, iNOS and COX-2. Collectively, these findings demonstrate the neuroprotective benefits of KF administration, supporting its potential as a dietary intervention for dysmetabolic-related neurodegenerative disorders.

## 1. Introduction

Overnutrition and modern diets containing high proportions of saturated fat are amongst the major factors responsible for the development of low-grade, systemic, chronic inflammation, hyperglycaemia and dyslipidaemia [1]. These conditions may eventually lead to insulin resistance (IR), a reduced ability of an organism to mount a normal and coordinated glucose lowering response via tissue-autonomous and crosstalk-dependent mechanisms [2]. If established, IR predisposes affected and susceptible subjects to a cluster of metabolic disorders such as type II Diabetes Mellitus (T2DM) and cardiovascular diseases [3].

Insulin and its receptors are widely expressed within the central nervous system, where they play key roles in controlling peripheral glucose metabolism, supporting cognition, enhancing neuron outgrowth and modulating the release and uptake of neurotransmitters [4].

Interestingly, recent studies started to recognize IR as a risk factor also for neurodegenerative conditions such as Alzheimer’s disease (AD) and other cognitive disorders [5,6]. Coherently, over 80% of AD patients have T2DM or abnormal serum glucose levels, suggesting that the pathogenic mechanisms of IR and AD might well overlap [7]. At this regard, IR has been shown to play a crucial role in the self-feeding cycle between chronic neuroinflammation and oxidative stress that underlies the development of the two hallmarks of AD i.e., extracellular amyloid-β (Aβ) plaques and intracellular neurofibrillary tangles (NFT) [6].

One of the most relevant molecular events underlying the AD-associated IR is the dysfunctional phosphorylation of the insulin receptor substrate (IRS)-1 by specific serine kinases [8,9,10,11]. These enzymes are activated through the action of selected, Aβ-induced, proinflammatory cytokines (interleukin (IL)-1β, IL-6, and tumour necrosis factor α (TNF-α) by glial cells [9]. Through the interaction with neuronal receptors, these microglial mediators markedly increase the phosphorylation of IRS-1 at S312, S616 and S636, consistently decreasing the physiological insulin activation of IRS-1 [8]. Indeed, pSerIRS-1 inhibits insulin downstream signalling events leading to both AKT inhibition and GSK-3β activation. Remarkably, this latter event has been strongly associated to NFT formation [12]. Moreover, elevated levels of neural pSerIRS-1 have been reported in both AD cortex and hippocampus, significantly correlate with the Aβ deposition and are associated with cognitive decline [13,14].

Within this scenario, increasing lines of evidence suggest that improving metabolic impairments could be effective to both reduce AD progression and ameliorate cognitive function [15,16,17]. Coherently recent research started to evaluate if therapeutic strategies aimed to prevent or counteract brain IR might contribute to overcome or ameliorate neurodegeneration and cognitive impairment [18,19,20].

Within fruit and vegetables rich in phytochemicals, citrus fruits contain relevant concentrations of several phytochemicals, mainly flavonoids, exerting significant anti-inflammatory, anti-oxidative, neuroprotective and anti-dysmetabolic effects [21,22]. Moreover, citrus peel is enriched in other bioactive compounds which have been shown to prevent and/or counteract T2DM and neurodegeneration progression [23,24,25,26].

While the health benefits arising from the consumption of several citrus fruits are well documented [27,28], only a few studies examined the health potential of either Kumquat consumption or its phytochemical supplementation [29,30].

Kumquats are small citrus fruits, produced by the *Fortunella japonica* tree, containing an abundance of phenolic compounds, including flavonoids and reducing molecules that provide Kumquat with a great healthy potential [31]. Among these, limonene has been found to be the major component of the Kumquat peel essential oil while neoeriocitrin and poncirin characterize the ethanolic extract of the fruit [31]. Relevantly, Kumquat fruits (KF) can be eaten along with the peel. Thanks to this aspect, it has been suggested that more phytochemicals can be assumed through its consumption and, therefore, better health benefits can be obtained in comparison to citrus fruits ingestion without peel. Accordingly, the administration of either the whole KF powder or its ethanolic extract has been shown to exert hypoglycaemic effects in diabetic rats or in obese mice respectively [29,32]. Interestingly, the underlying mechanisms and the identification of the active compounds responsible for such activities have not been explored yet. Moreover, the effects derived from the whole fruit administration on neurological alterations related to metabolic dysfunctions have not been so far evaluated.

In the light of the interconnections between neurodegenerative diseases and IR and taking into account the anti-oxidative, anti-inflammatory and anti-dysmetabolic potential of KF, the present work has evaluated whether and how the supplementation with the whole KF can counteract the brain IR-associated neurodegeneration, in an animal model with metabolic impairment and central neuropathological conditions, i.e., the high-fat, diet- (HFD) fed mouse.

## 2. Results

### 2.1. Metabolic Parameters

**Figure 1:**
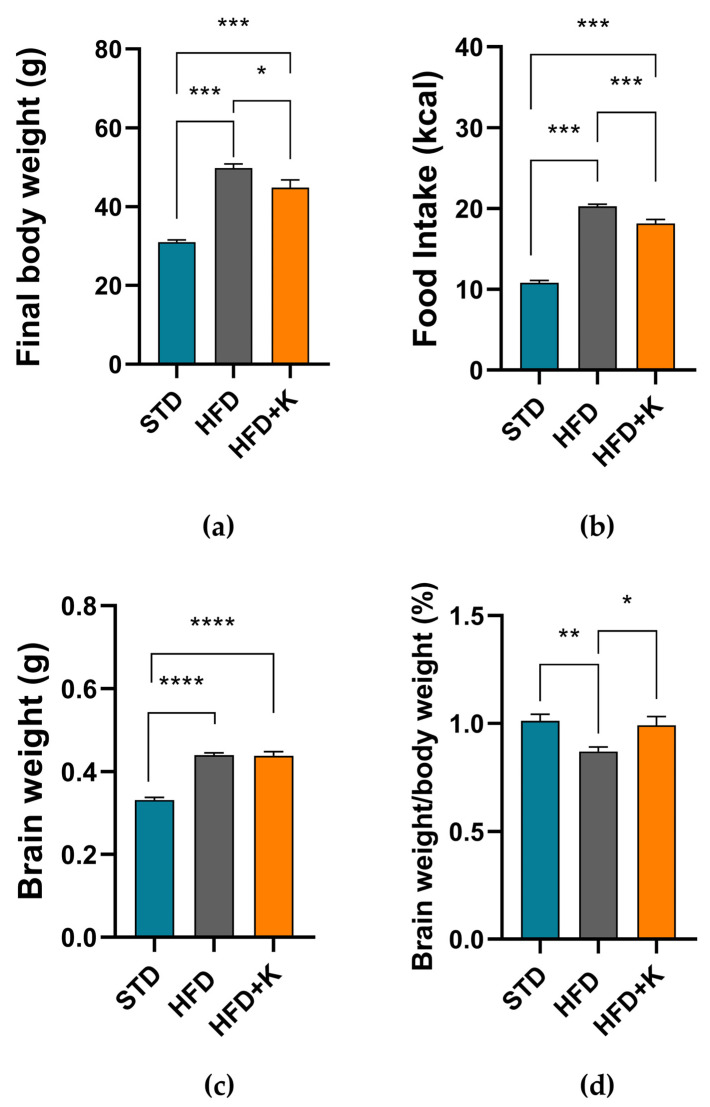
Evaluation of KF administration on weight gain and feeding behaviour. Body weight of the animals at the end of the study (**a**). Daily food intake (Kcal/*die*) (**b**). Brain weight immediately after sacrifice (**c**). Weight ratio brain/body expressed as a percentage (**d**). Histograms labelled with special characters are statistically significant (Anova one-way, followed by Tukey post hoc test). * = *p* < 0.05, ** *p* < 0.01; *** = *p* < 0.001, **** *p* < 0.0001.

After 24 weeks on HFD, mice showed a final body weight significantly higher compared to standard diet- (STD) fed mice. Interestingly, the group supplemented with KF (HFD+K) had both a final body weight and a daily energy intake significantly lower than the HFD positive controls (Figure 1a). Remarkably, we also observed that the brain weights and the brain/body weight ratio were significantly higher in the HFD fed mice compared to STD group, suggesting HFD-induce brain atrophy (Figure 1c,d). However, in HFD+K mice the brain/body weight ratio was similar to that observed in STD mice.

**Figure 2:**
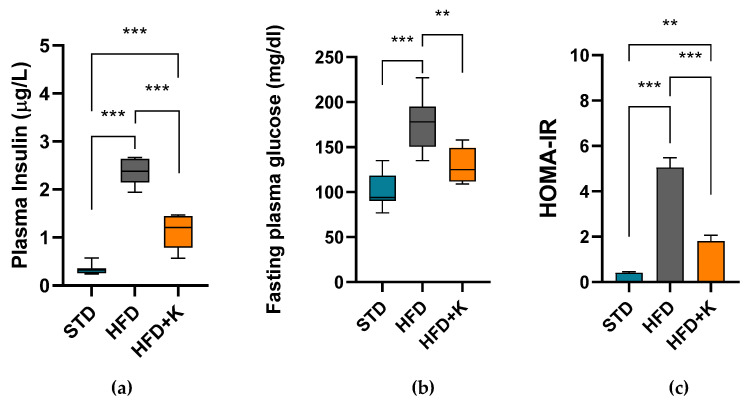
Evaluation of KF administration on the HFD-induced insulin resistance. Plasma insulin concentration (**a**) and fasting glycaemia (**b**) were utilized to calculate HOMA-IR, index of insulin resistance (**c**). Boxes or histograms labelled with special characters are statistically significant (Anova one-way, followed by Tukey post hoc test). ** = *p* < 0.01, *** = *p* < 0.001.

HFD obese mice also exhibited an impairment of glucose metabolism, as showed by the high plasma glucose and insulin levels and the increased Homeostatic Model Assessment for Insulin Resistance (HOMA-IR) index in comparation with STD mice. On the contrary, the HFD+K mice had plasma glucose and insulin concentration significantly lower than HFD mice and presented a clear improvement of insulin resistance as showed by the significantly reduced HOMA-IR (Figure 2).

### 2.2. KF Supplementation Reduces HFD-Induced Apoptosis in the Brain Cortex

**Figure 3:**
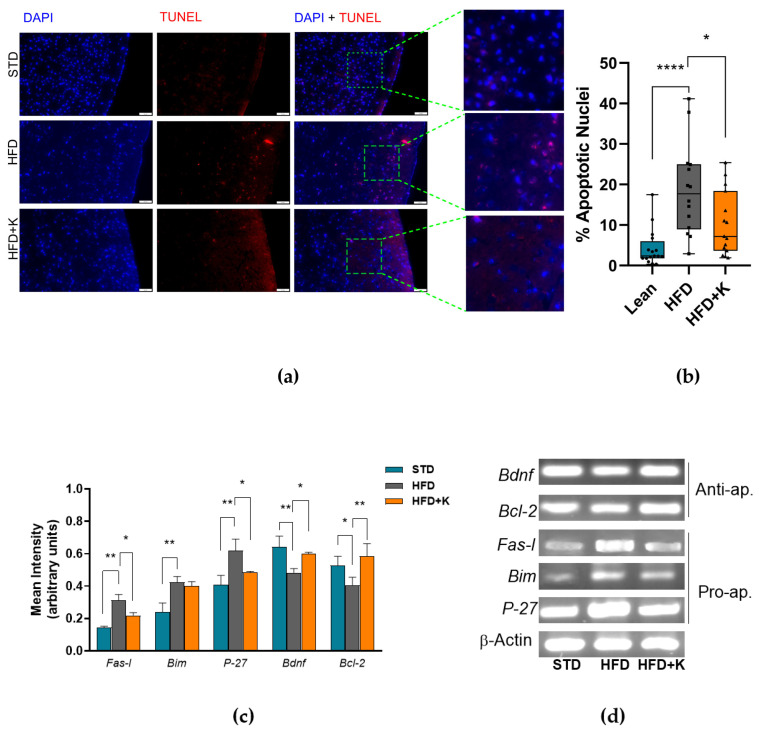
Evaluation of KF administration on the HFD-induced brain apoptosis. Representative images of TUNEL assays performed in coronal brain slices focusing on superficial cortices; scale bar: 50 µm (**a**). Percentage of TUNEL-positive (apoptotic) cells (**b**). Boxes labelled with special characters are statistically significant (Anova one-way, followed by Tukey post hoc test). * = *p* < 0.05, **** = *p* < 0.001. Histograms represent mean intensity of PCR-amplified DNA bands separated through electrophoresis on agarose gel (**c**). Representative DNA bands from agarose gel electrophoresis (**d**). Histograms labelled with special characters are statistically significant (Anova one-way, followed by Tukey post hoc test) Data are mean values ± S.E.M. * = *p* < 0.05, ** = *p* < 0.01. Anti-ap. = Anti-apoptotic; Pro-ap. = Pro apoptotic.

The effects of Kumquat consumption on the HFD-induced brain damage were investigated by TUNEL analysis which detects neuronal DNA fragmentation. Notwithstanding the high biological heterogeneity of the samples analysed, the TUNEL assay revealed significantly increased number of apoptotic neurons in the superficial cerebral cortex of HFD mice in comparation with STD mice or HFD+K mice (Figure 3a,b).

Relevantly, these data were paralleled by the PCR analysis of the expression of key genes involved in the regulation of the apoptotic process. Indeed, as shown in Figure 3c,d, the overexpression of the pro-apoptotic genes (*Fas-l*, *P-27*) and the downregulation of anti-apoptotic genes (*Bcl-2* and *Bdfn*) observed in the brains of HFD mice compared to STD mice, returned near to the normal expression levels in the brain of HFD-K mice.

### 2.3. Exploration of KF Influence on Genes Related to Alzheimer’s Disease

**Figure 4:**
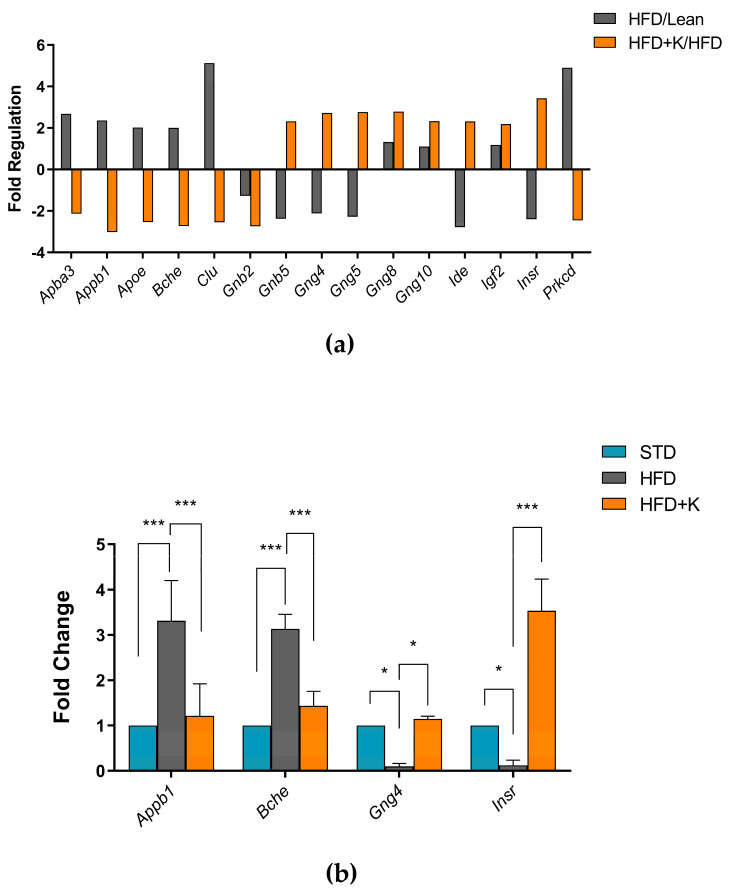
Evaluation of KF administration on the expression of AD-related genes. Main results of RT^2^ Profiler™ PCR Array for Alzheimer’s Disease (**a**). Results of RT-PCR used to confirm RT^2^ Profiler™ results (**b**). Histograms labelled with special characters are statistically significant (Anova one-way, followed by Tukey post hoc test). Data are mean values ± S.E.M. * = *p* < 0.05; *** = *p* < 0.01.

The Alzheimer’s Disease RT^2^ Profiler PCR Array analysis was used to analyse the expression variation of 84 genes associated with onset and progression of AD in the differently fed mice. We focused on the gene expression levels that were up or downregulated by more than two-fold among the different groups of mice. In the whole brain of HFD+K mice, we identified significant changes in the expression of fifteen genes compared to obese controls. Specifically, genes involved in neuronal cell signalling (*Gnb5*, *Gng4*, *Gng5*, *Gng8*, *Gng10*) and insulin regulation (*Ide*, *Igf2*, *Insr*) were upregulated. Conversely, genes associated with β-amyloid formation (*Apba3*, *Appb1*, *Apoe*, *Gnb2*, *Prkcd*), acetylcholine degradation (*Bche)*, and apoptosis (*Clu*) were downregulated in the HFD+K mice compared to the HFD group (Figure 4). No statistical difference in the expression of the above-reported genes between the STD and HFD+K groups was found (Supplementary Table S1).

To confirm the reliability of the gene expression profiling obtained from the RT^2^ Profiler PCR Array, we performed qRT-PCR analysis on four differentially expressed genes. Specifically, we selected *Appb1*, *Bche*, *Gng4* and *Insr* which showed significant upregulation or downregulation in HFD+K mice compared to HFD. The qRT-PCR results were consistent with the microarray data, confirming the observed trends in gene expression changes (Figure 4). The expression levels of *Gng4* and *InsR* were significantly increased, whereas *Appb1* and *Bche* showed a marked reduction in HFD+K mice compared to HFD mice.

### 2.4. KF Supplementation Ameliorates the HFD-Induced Dysfunction of the Insulin Signalling Axis in Brain Cortex

**Figure 5:**
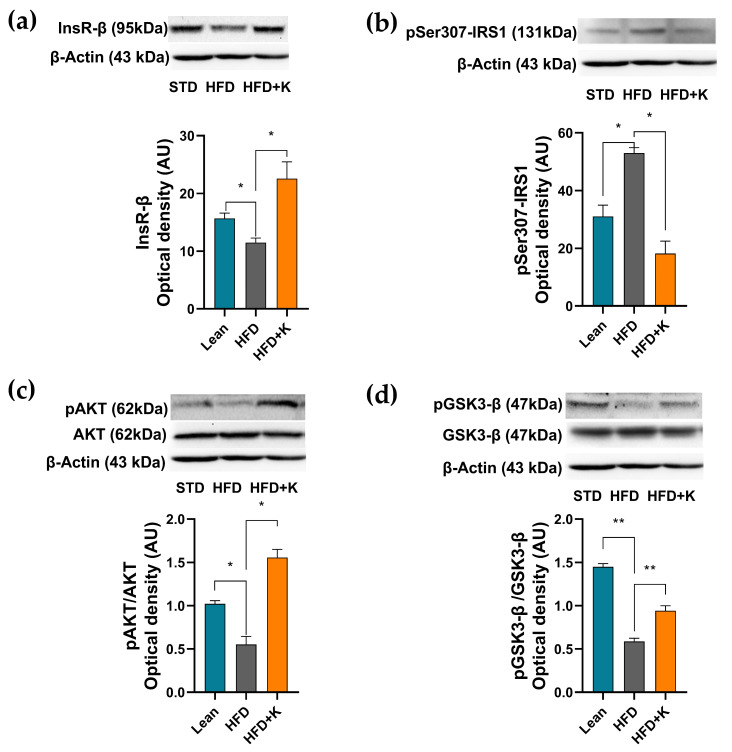
Evaluation of KF administration on the HFD-induced impairment of brain insulin signal transduction pathway. Representative blots and densitometric levels of InsR-β (**a**), inactive pSer307-IRS1 (**b**), pAKT/AKT (**c**) and pGSK3-β/GSK3-β (**d**). Histograms labelled with special characters are statistically significant (Anova one-way, followed by Tukey post hoc test). Data are mean values ± S.E.M. * = *p* < 0.05; ** = *p* < 0.01.

Given the ability of KF to counteract both peripheral IR and neuro-apoptosis in our experimental system, we next evaluated if these effects were associated with an improvement of brain insulin signalling. To this aim, the expression levels of a series of key proteins involved in the brain insulin signal transduction pathway were assessed. As shown in Figure 5a, mice subjected to a HFD regimen exhibited a significant decrease in the expression level of the insulin receptor subunit β (Ins-Rβ) in comparison with the STD group. Such a reduction was remarkably inhibited with KF supplementation, that increased Ins-Rβ levels above the control ones (Figure 5a). The investigation then proceeded by evaluating the effects of KF administration on the HFD-induced inactivation of IRS-1, by assessing pSerIRS-1 expression levels. As shown in Figure 5b our results demonstrated how HFD significantly increased pSer^307^IRS-1 levels with respect to the STD group and, relevantly, how KF supplementation was able to counteract this increase, reducing its levels below the control values. Given this evidence, we next investigated the phosphorylative state of AKT, which is dependent on the IRS-1 activation state, in our system. As expected, the HFD regimen induced a significant reduction of the p-AKT/AKT ratio with respect to the STD group (Figure 5c). Remarkably, KF supplementation significantly increased this ratio, well above the control levels. In the light of the ability of AKT to phosphorylate and thus inhibit GSK3-β, we next evaluated the activation state of this latter enzyme by assessing the p-GSK3-β/GSK3-β ratio. Coherently with the above-reported results, the HFD group exhibited decreased levels of p-GSK3-β/GSK3-β ratio that was significantly increased by KF supplementation (Figure 5d).

### 2.5. KF Lowers HFD-Linked Neuroinflammation

**Figure 6:**
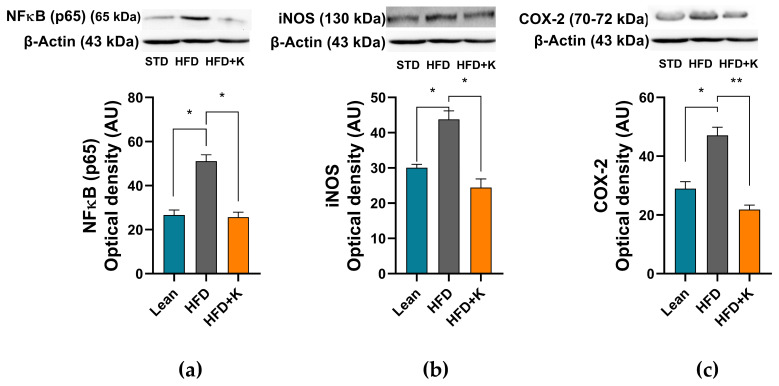
Evaluation of KF administration on the HFD-induced neuroinflammation. Representative blots and densitometric levels of NF-κB (**a**), iNOS (**b**) and COX-2 (**c**). Histograms labelled with special characters are statistically significant (Anova one-way, followed by Tukey post hoc test). Data are mean values ± S.E.M. (n = 8/group). * = *p* < 0.05; ** = *p* < 0.01.

Considering the remarks in the literature highlighting the role of inflammation in the development of IR and its role in the inhibition of IRS-1 though serine phosphorylation [11], we, then, investigated the involvement of pro-inflammatory events in our model. Through western blot analysis we found a remarkable overexpression of NF-κB in HFD group with respect to the STD one, hinting at a state of neuroinflammation. Remarkably, KF administration was able to counteract NF-κB overexpression bringing its levels back to control values (Figure 6a). Coherently, our results also showed how the expression of two key NF-κB downstream proteins i.e., COX-2 and iNOS were significantly increased in the HFD group and reduced to control level by KF supplementation (Figure 6b,c).

## 3. Discussion

This research aimed to evaluate the efficacy of KF supplementation to modulate two key and intertwined molecular processes underlying both AD and T2DM, i.e., the disruption of brain insulin signalling and the establishment of a chronic inflammatory state. Our findings reveal, for the first time, that KF administration significantly mitigates the HFD-induced neurodegeneration. Relevantly, we here demonstrate that these effects are associated to the KF ability to ameliorate brain insulin signalling and to reduce neuroinflammation.

A wealth of consolidated evidence indicates that HFD feeding induces a rapid reprogramming of systemic metabolism, leading to a marked weight gain and to an increased food intake. A previous study has demonstrated that an ethanolic extract from KF reduced body weight gain in HFD-fed mice after seven weeks of treatment [29]. In line with this evidence, we here report for the first time that also the whole KF administration effectively counteracts HFD-induced weight gain. Moreover, and unlike the previous findings on the ethanolic extract of KF, our results demonstrate that KF significantly reduced also the food intake. As a whole these findings highlight a better control of the HFD-induced obesogenic effects by the whole fruit over its ethanolic extract. Relevantly, and from a nutritional perspective, this evidence may also build a rationale to suggest the KF consumption in obesity-related conditions.

Consistently with a previous study showing the KF ability to improve glucose dysmetabolism in diabetic rats fed a high-fat, high-cholesterol diet [32], our results clearly demonstrate that KF administration markedly reduced fasting plasma glucose levels to control values. More importantly, we here report for the first time that KF treatment also significantly lowers plasma insulinemia and ameliorates IR. These results reveal a novel beneficial effect for KF and suggest a new mechanism through which this fruit may counteract the development of IR-related metabolic disorders.

In line with the peripheral, anti-dysmetabolic effects of either KF or its ethanolic extract [29,32], our study clearly shows the ability of KF to counteract HFD-induced neurodegeneration. Indeed, we here demonstrated that KF administration increased the brain-to-body weight ratio and reduced the apoptotic nuclei in the cortex. These findings align with the neuroprotective effects of other citrus fruits in dysmetabolic conditions. At the molecular level, we demonstrated that these effects were associated with a downregulation of the pro-apoptotic genes (*FAS-L*, *BIM* and *P27*) and an upregulation of the anti-apoptotic ones (*BDNF* and *BCL-2*). Interestingly, the inhibition of the HFD-induced neuroapotosis by KF, is coherent with the phytochemical fingerprint of KF, rich in limonene, neoeriocitrin and poncirin, repeatedly reported to counteract the apoptotic process also within the CNS [33].

AD and HFD-induced neurodegeneration share overlapping pathological features and underlying mechanisms [6,15,16,17]. Coherently with our results showing the ability of KF to exert neuroprotective effects mitigating HFD-induced neuroapoptosis we here show for the first time that its administration upregulates key genes involved in neuronal cell signalling and development (Gnb5 and Gng5) [34,35], cognitive performances (Gng4) [36], learning and memory (Gng8) [37] and insulin regulation (Ide, Igf2, InsR) [38,39]. Conversely, KF administration downregulates the expression of genes associated with β-amyloid formation (Apba3, Apbb1, Apoe, Gnb2, Prkcd) [40,41,42,43,44] butrylcholine degradation (Bche) [45] and apoptotic pathways (Clu) [46] with respect to the HFD group. While further demonstrating the ability of KF to positively modulate the expression of specific genes involved in neuronal cell death, our data also suggest new investigations to explore the potential efficacy of KF to enhance learning, memory and cognitive functions and to counteract neurodegenerative processes in terms of β-amyloid formation.

As previously discussed, brain IR has repeatedly been suggested as a mechanistic link between MetS and AD [5,47,48,49]. Impairment of four key mediators of insulin signalling, i.e., InsR, IRS-1, AKT, and GSK-3β, exerts a major role in the development of IR in the brain [6].

InsR levels on the cell membrane within the CNS are markedly reduced in dysmetabolism-associated neurodegenerative diseases [50]. Our findings, in line with the above-described ability of KF to upregulate the expression of selected genes involved in insulin regulation (Ide, Igf2, InsR), clearly demonstrate that KF administration significantly counteracts also the HFD-induced downregulation of InsR protein expression. While assessing for the first time the ability of KF to positively modulate InsR expression within the CNS our study also suggests the potential for KF to ameliorate brain IR. In addition, this result may foster further research to evaluate whether KF phytochemicals, e.g., neoeriocitrin and poncirin may account for this effect.

As previously stated, dysmetabolism-induced deposition of Aβ oligomers within the CNS can disrupt insulin signalling by over-activating IRS-1 serine kinases. This event can result in an excessive serine phosphorylation of IRS-1 and in the subsequent weakening of its function. In line with other published evidence [51,52], our results show that HFD significantly impairs brain insulin sensitivity, evaluated from the increase of p-serIRS-1 levels, [8,53]. Remarkably, in our system KF administration markedly reduces the HFD-induced IRS-1 phosphorylation at ser307. In the light of the key role exerted by p-serIRS-1 in the neurodegenerative processes [49], this evidence might provide a mechanistic basis the observed neuroprotective effects exerted by KF. Relevantly, this is the first study to evaluate the effect of KF on the IRS-1 s307 in an in vivo model of neurodegeneration.

AKT is known to act downstream of IRS-1 activation [54]. Notably and coherently with KF ability to decrease p-serIRS-1 levels, its administration effectively inhibited the HFD-induced reduction of p-AKT, increasing its levels well above the control values. Our findings may suggest a potential regulatory mechanism through which KF, by increasing p-AKT levels, might improve brain IR in response to prolonged HFD exposure. Moreover, considering the critical role of AKT as a regulator enzyme where metabolic and neurotrophic signals converge [4,55], its upregulation, induced by KF administration, might also well explain the anti-apoptotic and neuroprotective effects reported in our current study.

In dysmetabolism-associated neurodegenerative conditions the multifaceted GSK-3β is activated through a mechanism involving an increase of its unphosphorylated form [6,12,56,57,58]. In this scenario, previous studies have demonstrated that a decrease of p-Akt levels may results in the decrease in the p-GSK-3β and therefore in its activation. Consistent with this evidence and in line with the ability of HFD to inhibit AKT phosphorylation, our study showed that the HFD-induced reduction of pAKT was associated with an increase of the unphosphorylated, active GSK-3β activity. Remarkably, we here demonstrate for the first time that these effects were significantly attenuated by KF supplementation that was able to increase the levels of p-GSK-3β inactive form. As the active, unphosphorylated form of GSK-3β is deeply involved in tau hyper-phosphorylation and therefore in the neurodegenerative processes, our findings appear of interest as they may suggest the inactivation of GSK-3β by KF as a molecular event underlying its neuroprotective effects.

Metabolic syndrome and brain IR contribute to neuroinflammation through the induction of a systemic low-grade inflammatory state and the activation of pro-inflammatory signalling pathways [59]. Neuroinflammation, in turn, exacerbates AD neuro-pathology by multiple mechanisms including the induction of synaptic failure, inhibition of hippocampal neurogenesis and the promotion of neuronal apoptosis [48]. Within this scenario NF-κB has been identified to play a major role in the neuronal IRS-1 inhibition, by promoting the synthesis of pro-inflammatory cytokines and enzymes through Aβ-induced microglial activation [60]. Our results clearly demonstrating the capacity of KF to mitigate the HFD-induced NF-κB activation are noteworthy, as they might provide a mechanistic basis for its protective effects against HFD-induced neuroinflammation, IR and neurodegeneration. Notably, KF phytochemicals, including limonene and poncirin have been demonstrated to exert significant anti-neuroinflammatory effects by counteracting NF-κB activation or overexpression [33,61,62,63]. Along these lines, the currently observed neuroprotective potential of KF might well rely on the ability of its phytochemicals to modulate neuroinflammatory responses, thereby enhancing neuronal resilience against dysmetabolic stressors.

Among the downstream products of NF-κB activation iNOS and COX-2 play pivotal roles in the vicious cycle between IR, neuroinflammation and neurodegeneration [57,61]. Indeed, hypothalamic iNOS overexpression triggers brain IR and obesity through mechanisms involving the S-nitrosylation of insulin signalling-associated molecules such as IRS-1 and AKT [64]. Furthermore, HFD-induced aberrant iNOS expression in astrocytes promotes astrogliosis, exacerbate neuroinflammation, neurotoxicity and disrupt mitochondrial function [57,58,65]. On the other hand, under dysmetabolic conditions, COX-2 over-expression leads to an increase in prostaglandin production resulting in neurotoxicity, in the release of glutamate from astrocytes [66] and in the overproduction of pro-inflammatory cytokines, such as IL-1β, IL-6 and TNF-α [67]. Remarkably, our findings show that KF supplementation completely abrogates the HFD-induced iNOS overexpression and significantly reduces COX-2 levels, suggesting that by mitigating the dysregulation of these pro-inflammatory enzymes, KF may protect against HFD-induced brain IR, improve both central and systemic glucose metabolism and counteract obesity-related neuroinflammation.

## 4. Materials and Methods

### 4.1. Materials and Reagents

Unless otherwise specified, all reagents were bought from Sigma-Aldrich (Milan, Italy) at the highest level of purity available.

### 4.2. Animals and Diet

Male C57BL/6 mice were purchased from Envigo (S. Pietro al Natisone, Udine, Italy) and maintained in the Advanced Technologies Network (ATeN) center’s (Palermo, Italy) animal facility according to the European guidelines. The animals (n = 24, 4-weeks old) were housed (2 mice/cage) in a temperature-(23 ± 1 °C) and relative humidity-(55% ± 5%) controlled facility, under a 12-h light–dark cycle, according to the Italian legislative decree n. 26/2014 and the experiments were approved by the Ministry of Health (Rome, Italy; Authorization No. 46/2020-PR issued on 21 January 2020). After one week of acclimatization, the mice were randomly subdivided into three groups (n = 8/group) and assigned to specific diets: a standard diet (STD) (representing our negative control) containing protein 20.0%, fat 10.0%, carbohydrate 70.0%, *w*/*w*, and water (ref. 4RF25, Mucedola, Milan, Italy); a HFD, containing protein 20.0%, fat 60.0%, carbohydrate 20.0%, *w*/*w* (PF4215, Mucedola, Milan, Italy); a HFD supplemented with 5% Kumquat whole fruit (50 g of lyophilised Kumquat/Kg HFD) (Mucedola, Milan, Italy) (HFD+K) and isocaloric with the HFD diet of the second group. The animals were differently fed for 24 weeks, during which bodyweight and food intake were detected weekly. At the end of the experimental protocol, metabolic parameters were analysed, and the animals were sacrificed by cervical dislocation. Blood was immediately drawn by cardiac puncture and centrifuged at 3000 rpm for 15 min at 4 °C to obtain plasma that was stored at −80 °C until analysis. At the same time, brains were rapidly collected, weighted.

### 4.3. Metabolic Parameters

Fasting plasma glucose concentration was measured using a commercial glucometer (GlucoMen LX meter, Menarini, Florence, Italy) in overnight-fasted mice, by a drop of blood collected from the tail vein. Plasma insulin was determined by a mouse ELISA kit, according to manufacturer’s instructions (Alpco diagnostics, Salem, NH, USA) and homeostasis model assessment of insulin resistance (HOMA-IR) was calculated, as follow: serum glucose (mmol/L) × serum insulin (mU/L)/22.5.

### 4.4. Brain Tissue Preparation

The excised brains were dissected sagittally into two halves. One half was homogenized on ice using a Dounce homogenizer, then divided into aliquots (5 or 10 mg), rapidly frozen in liquid nitrogen and stored until further analysis. The other half brain was processed for histological assays. Thus, this portion was fixed in 4% formalin for 24 h, followed by dehydration in graded ethanol solutions (50%, 70%, 85%, 96%) for 5 min each, then embedded in paraffin overnight. Subsequently, sections of 5 μm thick were cut via an automatic microtome (Leica Biosystems, Buffalo Grove, IL, USA).

### 4.5. Terminal Deoxynucleotidyl Transferase Biotin-dUTP Nick End Labeling (TUNEL) Assay

Apoptosis was evaluated by TUNEL assay with a dedicated kit (Promega, Madison, WI, USA) as previously reported [47,68,69]. Briefly, brain samples were sliced into 5 µm coronal sections after being embedded in paraffin. Then, the deparaffinized slices were hydrated in a series of graded ethanol solutions (96%, 85%, 70%, and 50%) for 5 min each, washed twice in PBS and incubated with TUNEL working solution for 1 h at 37 °C. Diamidino-2-phenylindole (DAPI) (Invitrogen-Thermo Fisher Scientific, Waltham, MA, USA) solution was used as a nuclear staining. Slices were observed under a confocal fluorescence microscope (Leica Microsystems, Heidelberg, Germany) and images were acquired via the image analysis software Basic Research NIS Elements F 2.30 (Nikon, Florence, Italy). Apoptotic nuclei were identified based on TUNEL positivity (red fluorescence) with nuclear condensation and/or fragmentation. Counting was performed manually by two independent researchers, blinded to experimental group. For each section, five non-overlapping fields were randomly selected within the cerebral cortex, and apoptotic nuclei were counted. The results were expressed as the percentage of TUNEL-positive nuclei relative to the total number of DAPI-stained nuclei in the selected fields.

### 4.6. Semiquantitative Polymerase Chain Reaction Experiments

RNA was extracted from the brain tissue using the PureLink RNA Mini Kit (Invitrogen, Thermo Fisher Scientific, Waltham, MA, USA). Subsequently, cDNA was prepared by 2 ng of total RNA using High-Capacity cDNA Reverse Transcription Kit (Applied Biosystems, Waltham, MA, USA). Reverse Transcription Polymerase Chain Reaction (RT-PCR) was then performed to assess the expression of pro- and anti-apoptotic genes (*Fas-L*, *Bim*, *P27*, *Bcl-2*, *BDNF*), using primers listed in Table 1. Amplification cycles included denaturation (95 °C for 45 s), annealing (52 °C for 45 s) and extension (72 °C for 45 s) for 40 cycles. Subsequently, amplification products were visualized using E-Gel GelCapture (Thermo Fisher Scientific, Monza, Italy) after separation by agarose gel electrophoresis. Signal intensity of amplified products, corresponding to the expression levels of target genes, was analyzed using the E-Gel GelQuant Express software 4.1 (Thermo Fisher Scientific, Monza, Italy) and normalized to its respective *β-actin* signal intensity.

### 4.7. RT^2^ Profiler PCR Array

Gene expression changes in the brains of differently fed mice were assessed using the Mouse Alzheimer’s Disease RT^2^ Profiler PCR Array (Qiagen, Monza, Italy) in a 96-well plate format. Total RNA was extracted from STD, HFD, and HFD-K mouse brains using the RNeasy Microarray Tissue Mini Kit (Qiagen, Monza, Italy). Briefly, 35 mg of brain tissue were homogenized and lysed in QIAzol (a phenol-guanidine-based lysis buffer), followed by RNA purification with the RNeasy Mini Kit (Qiagen, Monza, Italy). RNA was quantified by spectrophotometry (260 nm absorbance) and its integrity verified via electrophoresis on a 1% agarose gel. cDNA was synthesized from 2 ng of RNA using the RT^2^ First Strand Kit (Qiagen, Monza, Italy) and subsequently amplified with the StepOne Real-Time PCR System (Applied Biosystems) using the RT^2^ Profiler PCR Array for Alzheimer’s disease (Qiagen, Monza, Italy), following the manufacturer’s instructions. Gene expression analysis was conducted using the relative quantification method (2^−ΔΔCt^). Gene expression was validated using quantitative real-time PCR (qPCR) with predesigned primers purchased from Qiagen (Hilden, Germany). Total RNA was extracted from whole brain tissue using the PureLink RNA Mini Kit (Invitrogen, Thermo Fisher Scientific, Waltham, MA, USA) according to the manufacturer’s instructions. Two nanograms of RNA were reverse transcribed into cDNA using the High-Capacity cDNA Reverse Transcription Kit (Applied Biosystems, Waltham, MA, USA). The synthesized cDNA was then amplified using the Applied Biosystems™ PowerUp™ SYBR™ Green Master Mix for qPCR (Thermo Fisher Scientific, Waltham, MA, USA) on a StepOne Real-Time PCR System (Applied Biosystems, Waltham, MA, USA). *β-actin* was used as the reference gene for normalization.

### 4.8. Western Blot Analysis

Tissues were homogenized on ice-cold buffer containing 50 mM Tris-HCl (pH 7.4), 150 mM NaCl, 1 mM EDTA, 1% Triton X-100, 24 mM sodium deoxycholate, 0.01% SDS, 10 mM sodium pyrophosphate, 100 mM sodium fluoride, 10 mM sodium orthovanadate, 1.5 μM aprotinin, 1 mM phenylmethanesulfonylfluoride (PMSF) and 2.1 μM leupeptin. Protein quantification from homogenate brain tissues was performed by Bradford assay (Quick Start™ Bradford 1x Dye Reagent, Bio-Rad, Milan, Italy), according to manufacturer’s instructions. Western blot analysis was performed as previously described [70] with minor alterations. Briefly, 35 µg of protein sample was resolved via SDS-PAGE on either 8%, or 10% or 12% acrylamide gels according to needs and blotted onto nitrocellulose membranes (Amersham Protran 0.45 NC nitrocellulose Western blotting membrane, Cytiva, Milan, Italy). Coloured protein molecular weight ladders were used to monitor the progress of protein electrophoresis, to assess the transfer efficiency and to control the molecular weight of the proteins to be evaluated (Amersham™, ECL™ Rainbow™ Marker-Full Range, VWR, Milan, Italy, ref. GERPN800E). After blocking for 2 h in 5% (*w*/*v*) skim milk, the membranes were incubated overnight at 4 °C in the presence of the primary antibodies shown in Table 2. The membranes were then incubated for 90 min at room temperature with anti-mouse or anti-rabbit IgG and HRP-conjugated secondary antibodies according to needs (Table 2). After exposure to ECL (Amersham, Milan, Italy) solution, specific chemiluminescent bands were detected via an iBright FL1500 (Thermo Fisher Scientific, Waltham, MA, USA) imaging system, and densitometric analysis was performed via FIJI (ImageJ 1.53f software, Laboratory for Optical and Computational Instrumentation-LOCI, University of Wisconsin, Madison, WI, USA) [71]. Protein expression levels were normalized to those of β-actin.

### 4.9. Statistical Analysis

The results are reported as mean ±SEM. Statistical analysis was performed by ANOVA, followed by Tukey’s post hoc test using Prism 6.0, GraphPad (San Diego, CA, USA). Results with a *p* value < 0.05 were considered statistically significant.

## 5. Conclusions

In conclusion, this study provides novel evidence that KF administration exerts significant neuroprotective effects, counteracting dysmetabolism-induced neuronal damage in the HFD model. From a mechanistic perspective, these effects are associated with KF ability to mitigate the disruption in the brain insulin signal transduction pathway and attenuate neuroinflammation. In the light of the strict interconnections between neurodegenerative diseases and IR, these findings appear particularly significant as they suggest a molecular explanation for the observed neuroprotective effects of KF. The limitations of the study are related to its preclinical value but at the same time our current evidence might well foster further studies in humans aimed to evaluate the potential of KF-based dietary interventions for dysmetabolic-related neurodegenerative disorders.

## Figures and Tables

**Table 1:** Oligonucleotide Primer Sequences for RT-PCR.

Gene	Forward Primer	Reverse Primer	T° Annealing
*Fas-l*	5′-CAAGTCCAACTCAAGGTCCATGCC-3′	5′-AGAGAGGCTCAGATACGTTTGAC-3′	58 °C
*Bim*	5′-GGAGGAGGCGGAGGATGAT-3′	5′-TCCTGTCTTGCGGTTCTGTC-3′	58 °C
*P 27*	5′-TGCGAGTGTCTAACGGGAG-3′	5′-GTTTGACGTCTTCTGAGGCC-3′	59 °C
*Bcl-2*	5′-ATGTGTGTGGAGAGCGTCAA-3′	5′-AGAGACAGCCAGGAGAAATCA-3′	47 °C
*bdnf*	5′-GGCTGACACTTTTGAGCACGTC-3′	5′-CTCCAAAGGCACTTGACTGCTG-3′	52 °C
*β-* *actin*	5′-CGGGATCCCCGCCCTAGGCACCAGGGT-3′	5′-GGAAATTCGGCTGGGGTGTTGAAGGTCTCAAA-3′	60 °C

**Table 2:** Primary/Secondary antibodies used for western blot analysis.

Protein	Manufacturer	Cat #	Host Organism	Molecular Weight
**Primary antibodies**				
AKT ^1^	Santa Cruz Biotechnologies (Milan, Italy)	SC-5298	Mouse	62 kDa
p-AKT ^1^	Sigma-Aldritch (Milan, Italy)	SAB4504331	Rabbit	62KDa
β-Actin	Santa Cruz Biotechnologies (Milan, Italy)	SC 47778	Mouse	43 kDa
COX-2 ^2^	Invitrogen (Milan, Italy)	35-8200	Mouse	72 kDa
GSK3 β ^3^	Santa Cruz Biotechnologies (Milan, Italy)	SC-377213	Mouse	47 kDa
p-GSK3 β ^3^	Santa Cruz Biotechnologies (Milan, Italy)	SC-373800	Mouse	47 kDa
iNOS ^4^	Invitrogen (Milan, Italy)	PA1-036	Rabbit	130 kDa
InsR-β ^5^	Santa Cruz Biotechnologies (Milan, Italy)	SC-57342	Mouse	95 kDa
pSer307-IRS1 ^6^	Merck (Milan, Italy)	SAB4504442	Rabbit	131 kDa
NF-κB ^7^	Santa Cruz Biotechnologies (Milan, Italy)	SC-8008	Mouse	65 kDa
**Secondary antibodies**				
IgG-HRP-Conjugated Anti-Mouse	Sigma-Aldrich (Milan, Italy)	A9044	Rabbit	/
IgG-HRP-Conjugated Anti-Rabbit	Sigma-Aldrich (Milan, Italy)	A0545	Goat	/

1: (p-) Protein Kinase B; 2: Cyclooxygenase 2; 3: (p-) Glycogen-Synthase Kinase 3 β; 4: inducible-Nitric Oxide Synthase; 5: Insulin Receptor subunit β; 6: Insulin Receptor Substrate 1; 7: Nuclear factor kappa-light-chain-enhancer of activated B cells.

## Data Availability

The raw data supporting the conclusions of this article will be made available by the authors on request.

## Supplementary Materials

The following supporting information can be downloaded at: https://www.mdpi.com/article/10.3390/ijms26073077/s1.

## Abbreviations

The following abbreviations are used in this manuscript:

Aβ	Amyloid-β
AD	Alzheimer’s Disease
HFD	High-Fat Diet
HFD+K	High Fat Diet supplemented with Kumquat fruit
HOMA-IR	Homeostatic Model Assessment for Insulin Resistance
IL1β	InterLeukin 1 β
Ins-Rβ	Insulin Receptor subunit β
IR	Insulin Resistance
IRS1	Insulin Receptor Substrate 1
KF	Kumquat Fruit
NFT	NeuroFibrillary Tangles
qPCR	quantitative real-time PCR
STD	Standard Diet
RT-PCR	Reverse Transcription Polymerase Chain Reaction
T2DM	Type 2 Diabetes Mellitus
TNF-α	Tumor Necrosis Factor α
TUNEL	Terminal deoxynucleotidyl transferase biotin-dUTP Nick End Labeling

## References

[1] Barbaresko J., Koch M., Schulze M.B., Nöthlings U. (2013). Dietary Pattern Analysis and Biomarkers of Low-Grade Inflammation: A Systematic Literature Review. Nutr. Rev..

[2] Lee S.H., Park S.Y., Choi C.S. (2022). Insulin Resistance: From Mechanisms to Therapeutic Strategies. Diabetes Metab. J..

[3] Caussy C., Aubin A., Loomba R. (2021). The Relationship Between Type 2 Diabetes, NAFLD, and Cardiovascular Risk. Curr. Diab. Rep..

[4] Chen W., Cai W., Hoover B., Kahn C.R. (2022). Insulin Action in the Brain: Cell Types, Circuits, and Diseases. Trends Neurosci..

[5] Sędzikowska A., Szablewski L., Rostagno A.A., Baranowska-Bik A., Orzechowski A. (2021). Insulin and Insulin Resistance in Alzheimer’s Disease. Int. J. Mol. Sci..

[6] Zheng M., Wang C., Hu M., Li Q., Li J., Quan S., Zhang X., Gu L. (2024). Research Progress on the Association of Insulin Resistance with Type 2 Diabetes Mellitus and Alzheimer’s Disease. Metab. Brain Dis..

[7] Zhao W.Q., Townsend M. (2009). Insulin Resistance and Amyloidogenesis as Common Molecular Foundation for Type 2 Diabetes and Alzheimer’s Disease. Biochim. Biophys. Acta.

[8] Woo J.R., Bae S.H., Wales T.E., Engen J.R., Lee J., Jang H., Park S.Y. (2024). The Serine Phosphorylations in the IRS-1 PIR Domain Abrogate IRS-1 and IR Interaction. Proc. Natl. Acad. Sci. USA.

[9] Ono H. (2019). Molecular Mechanisms of Hypothalamic Insulin Resistance. Int. J. Mol. Sci..

[10] Bhattacharyya S., Feferman L., Tobacman J.K. (2015). Carrageenan Inhibits Insulin Signaling through GRB10-Mediated Decrease in Tyr(P)-IRS1 and through Inflammation-Induced Increase in Ser(P)307-IRS1. J. Biol. Chem..

[11] Copps K.D., White M.F. (2012). Regulation of Insulin Sensitivity by Serine/Threonine Phosphorylation of Insulin Receptor Substrate Proteins IRS1 and IRS2. Diabetologia.

[12] Sayas C.L., Ávila J. (2021). GSK-3 and Tau: A Key Duet in Alzheimer’s Disease. Cells.

[13] Rong J., Wang Y., Liu N., Shen L., Ma Q., Wang M., Han B. (2024). Chronic Stress Induces Insulin Resistance and Enhances Cognitive Impairment in AD. Brain Res. Bull..

[14] Talbot K., Wang H.Y., Kazi H., Han L.Y., Bakshi K.P., Stucky A., Fuino R.L., Kawaguchi K.R., Samoyedny A.J., Wilson R.S. (2012). Demonstrated Brain Insulin Resistance in Alzheimer’s Disease Patients Is Associated with IGF-1 Resistance, IRS-1 Dysregulation, and Cognitive Decline. J. Clin. Investig..

[15] Solch R.J., Aigbogun J.O., Voyiadjis A.G., Talkington G.M., Darensbourg R.M., O’Connell S., Pickett K.M., Perez S.R., Maraganore D.M. (2022). Mediterranean Diet Adherence, Gut Microbiota, and Alzheimer’s or Parkinson’s Disease Risk: A Systematic Review. J. Neurol. Sci..

[16] Grant W.B., Blake S.M. (2023). Diet’s Role in Modifying Risk of Alzheimer’s Disease: History and Present Understanding. J. Alzheimer’s Dis..

[17] Stefaniak O., Dobrzyńska M., Drzymała-Czyż S., Przysławski J. (2022). Diet in the Prevention of Alzheimer’s Disease: Current Knowledge and Future Research Requirements. Nutrients.

[18] Hallschmid M. (2021). Intranasal Insulin for Alzheimer’s Disease. CNS Drugs.

[19] Farokhi Larijani S., Hassanzadeh G., Zahmatkesh M., Radfar F., Farahmandfar M. (2024). Intranasal Insulin Intake and Exercise Improve Memory Function in Amyloid-β Induced Alzheimer’s-like Disease in Rats: Involvement of Hippocampal BDNF-TrkB Receptor. Behav. Brain Res..

[20] AboEl-Azm Y.H., El-Samahy M., Hendi N.I., Arar A., Yasen N.S., Ramadan S., Zedan E.M., Al-dardery N.M., Khaity A. (2023). Safety and Efficacy of Intranasal Insulin in Patients with Alzheimer’s Disease: A Systematic Review and Meta-Analysis. J. Clin. Transl. Res..

[21] Wang Y., Liu X.J., Chen J.B., Cao J.P., Li X., Sun C. (2022). De Citrus Flavonoids and Their Antioxidant Evaluation. Crit. Rev. Food Sci. Nutr..

[22] Singh B., Singh J.P., Kaur A., Singh N. (2020). Phenolic Composition, Antioxidant Potential and Health Benefits of Citrus Peel. Food Res. Int..

[23] Matsuzaki K., Ohizumi Y. (2021). Beneficial Effects of Citrus-Derived Polymethoxylated Flavones for Central Nervous System Disorders. Nutrients.

[24] Azhar S., Sabahat R., Sajjad R., Nadeem F., Amjad A., Hafeez N., Nayab T., Wahid S., Tanweer A. (2023). Effect of Citrus Flavanones on Diabetes: A Systematic Review. Curr. Diabetes Rev..

[25] Hwang S.L., Shih P.H., Yen G.C. (2012). Neuroprotective Effects of Citrus Flavonoids. J. Agric. Food Chem..

[26] Gandhi G.R., Vasconcelos A.B.S., Wu D.T., Li H.B., Antony P.J., Li H., Geng F., Gurgel R.Q., Narain N., Gan R.Y. (2020). Citrus Flavonoids as Promising Phytochemicals Targeting Diabetes and Related Complications: A Systematic Review of In Vitro and In Vivo Studies. Nutrients.

[27] Pontifex M.G., Malik M.M.A.H., Connell E., Müller M., Vauzour D. (2021). Citrus Polyphenols in Brain Health and Disease: Current Perspectives. Front. Neurosci..

[28] Miles E.A., Calder P.C. (2021). Effects of Citrus Fruit Juices and Their Bioactive Components on Inflammation and Immunity: A Narrative Review. Front. Immunol..

[29] Tan S., Li M., Ding X., Fan S., Guo L., Gu M., Zhang Y., Feng L., Jiang D., Li Y. (2014). Effects of Fortunella Margarita Fruit Extract on Metabolic Disorders in High-Fat Diet-Induced Obese C57BL/6 Mice. PLoS ONE.

[30] Zeng H., Chen P., Chang Q., Zheng B., Zhang Y. (2019). Hypolipidemic Effect of Polysaccharides from Fortunella Margarita (Lour.) Swingle in Hyperlipidemic Rats. Food Chem. Toxicol..

[31] Lou S.N., Ho C.T. (2017). Phenolic Compounds and Biological Activities of Small-Size Citrus: Kumquat and Calamondin. J. Food Drug Anal..

[32] Al-Sayed H.M.A., Abdelaleem M.A., Shawky H.A. (2021). Physiochemical and Nutritional Evaluation of Whole Kumquat Fruits Powder and Its Protective Effect on Thyroid Hormones and Blood Sugar Levels in Diabetic Rats. Braz. J. Biol..

[33] Eddin L.B., Azimullah S., Jha N.K., Nagoor Meeran M.F., Beiram R., Ojha S. (2023). Limonene, a Monoterpene, Mitigates Rotenone-Induced Dopaminergic Neurodegeneration by Modulating Neuroinflammation, Hippo Signaling and Apoptosis in Rats. Int. J. Mol. Sci..

[34] Zhang J., Pandey M., Awe A., Lue N., Kittock C., Fikse E., Degner K., Staples J., Mokhasi N., Chen W. (2024). The Association of GNB5 with Alzheimer Disease Revealed by Genomic Analysis Restricted to Variants Impacting Gene Function. Am. J. Hum. Genet..

[35] Grimm M.O.W., Lauer A.A., Grösgen S., Thiel A., Lehmann J., Winkler J., Janitschke D., Herr C., Beisswenger C., Bals R. (2019). Profiling of Alzheimer’s Disease Related Genes in Mild to Moderate Vitamin D Hypovitaminosis. J. Nutr. Biochem..

[36] Bonham L.W., Evans D.S., Liu Y., Cummings S.R., Yaffe K., Yokoyama J.S. (2018). Neurotransmitter Pathway Genes in Cognitive Decline During Aging: Evidence for GNG4 and KCNQ2 Genes. Am. J. Alzheimer’s Dis. Other Demen..

[37] Lee H.J., Choi T.I., Kim Y.M., Lee S., Han B., Bak I.S., Moon S.A., Yu D.Y., Shin K.S., Kwon Y.K. (2021). Regulation of Habenular G-Protein Gamma 8 on Learning and Memory via Modulation of the Central Acetylcholine System. Mol. Psychiatry.

[38] Lee Y., Kim Y.H., Park S.J., Huh J.W., Kim S.H., Kim S.U., Kim J.S., Jeong K.J., Lee K.M., Hong Y. (2014). Insulin/IGF Signaling-Related Gene Expression in the Brain of a Sporadic Alzheimer’s Disease Monkey Model Induced by Intracerebroventricular Injection of Streptozotocin. J. Alzheimer’s Dis..

[39] Vepsäläinen S., Helisalmi S., Mannermaa A., Pirttilä T., Soininen H., Hiltunen M. (2009). Combined Risk Effects of IDE and NEP Gene Variants on Alzheimer Disease. J. Neurol. Neurosurg. Psychiatry.

[40] Du Y., Guo T., Hao Y., Li C., Tang L., Li X., Zhang X., Li L., Yao D., Xu X. (2024). PKCδ Serves as a Potential Biomarker and Therapeutic Target for Microglia-Mediated Neuroinflammation in Alzheimer’s Disease. Alzheimer’s Dement..

[41] Kitanaka J., Wang X.B., Kitanaka N., Hembree C.M., Uhl G.R. (2001). Genomic Organization of the Murine G Protein Beta Subunit Genes and Related Processed Pseudogenes. DNA Seq..

[42] Reiss A.B., Housny M., Gulkarov S., Hossain T., Locke B., Srivastava A., Pinkhasov A., Gomolin I.H., Wisniewski T., De Leon J. (2024). Role of Apolipoprotein E in Alzheimer’s Disease Pathogenesis, Prognosis and Treatment. Discov. Med..

[43] Bonifácio M.J., Sousa F., Aires C., Loureiro A.I., Fernandes-Lopes C., Pires N.M., Palma P.N., Moser P., Soares-da-Silva P. (2020). Preclinical Pharmacological Evaluation of the Fatty Acid Amide Hydrolase Inhibitor BIA 10-2474. Br. J. Pharmacol..

[44] Kant S., Stopa E.G., Johanson C.E., Baird A., Silverberg G.D. (2018). Choroid Plexus Genes for CSF Production and Brain Homeostasis Are Altered in Alzheimer’s Disease. Fluids Barriers CNS.

[45] Ghosh S., Das B., Jana S., Singh K.O., Sharma N., Mukherjee P.K., Haldar P.K. (2025). Mechanistic Insight into Neuroprotective Effect of Standardized Ginger Chemo Varieties from Manipur, India in Scopolamine Induced Learning and Memory Impaired Mice. Metab. Brain Dis..

[46] Laslo A., Laslo L., Arbănași E.M., Ujlaki-Nagi A.A., Chinezu L., Ivănescu A.D., Arbănași E.M., Cărare R.O., Cordoș B.A., Popa I.A. (2024). Pathways to Alzheimer’s Disease: The Intersecting Roles of Clusterin and Apolipoprotein E in Amyloid-β Regulation and Neuronal Health. Pathophysiology.

[47] Nuzzo D., Picone P., Baldassano S., Caruana L., Messina E., Gammazza A.M., Cappello F., Mulè F., Carlo M. (2015). Di Insulin Resistance as Common Molecular Denominator Linking Obesity to Alzheimer’s Disease. Curr. Alzheimer Res..

[48] Vinuesa A., Pomilio C., Gregosa A., Bentivegna M., Presa J., Bellotto M., Saravia F., Beauquis J. (2021). Inflammation and Insulin Resistance as Risk Factors and Potential Therapeutic Targets for Alzheimer’s Disease. Front. Neurosci..

[49] Bosco D., Fava A., Plastino M., Montalcini T., Pujia A. (2011). Possible Implications of Insulin Resistance and Glucose Metabolism in Alzheimer’s Disease Pathogenesis. J. Cell. Mol. Med..

[50] Petrov D., Pedrós I., Artiach G., Sureda F.X., Barroso E., Pallàs M., Casadesús G., Beas-Zarate C., Carro E., Ferrer I. (2015). High-Fat Diet-Induced Deregulation of Hippocampal Insulin Signaling and Mitochondrial Homeostasis Deficiences Contribute to Alzheimer Disease Pathology in Rodents. Biochim. Biophys. Acta (BBA)-Mol. Basis Dis..

[51] Turdi S., Ge W., Hu N., Bradley K.M., Wang X., Ren J. (2013). Interaction between Maternal and Postnatal High Fat Diet Leads to a Greater Risk of Myocardial Dysfunction in Offspring via Enhanced Lipotoxicity, IRS-1 Serine Phosphorylation and Mitochondrial Defects. J. Mol. Cell. Cardiol..

[52] Zhao T., Li Q., Wang X., Tang B., Zhang X., Yu H., Li Z. (2024). Time-Dependent Effects of High-Fat Diet on Cognition and Cerebral Insulin Signaling: Window for Recovery and Potential Therapeutic Target. Mech. Ageing Dev..

[53] Zheng M., Wang P. (2021). Role of Insulin Receptor Substance-1 Modulating PI3K/Akt Insulin Signaling Pathway in Alzheimer’s Disease. 3 Biotech.

[54] Petersen M.C., Shulman G.I. (2018). Mechanisms of Insulin Action and Insulin Resistance. Physiol. Rev..

[55] Duronio V. (2008). The Life of a Cell: Apoptosis Regulation by the PI3K/PKB Pathway. Biochem. J..

[56] Shareena G., Kumar D., Thorat N. (2023). Exploring the Diverse Roles of GSK-3β Kinase in Alzheimer’s Disease. Deciphering Drug Targets for Alzheimer’s Disease.

[57] Katashima C.K., Silva V.R.R., Lenhare L., Marin R.M., Carvalheira J.B.C. (2017). INOS Promotes Hypothalamic Insulin Resistance Associated with Deregulation of Energy Balance and Obesity in Rodents. Sci. Rep..

[58] Patel B., New L.E., Griffiths J.C., Deuchars J., Filippi B.M. (2021). Inhibition of Mitochondrial Fission and INOS in the Dorsal Vagal Complex Protects from Overeating and Weight Gain. Mol. Metab..

[59] Ali N.H., Al-Kuraishy H.M., Al-Gareeb A.I., Alexiou A., Papadakis M., Bahaa M.M., Alibrahim F., Batiha G.E.S. (2024). New Insight on the Potential Detrimental Effect of Metabolic Syndrome on the Alzheimer Disease Neuropathology: Mechanistic Role. J. Cell. Mol. Med..

[60] Block M.L., Zecca L., Hong J.S. (2007). Microglia-Mediated Neurotoxicity: Uncovering the Molecular Mechanisms. Nat. Rev. Neurosci..

[61] Kim J.B., Han A.R., Park E.Y., Kim J.Y., Cho W., Lee J., Seo E.K., Lee K.T. (2007). Inhibition of LPS-Induced INOS, COX-2 and Cytokines Expression by Poncirin through the NF-ΚB Inactivation in RAW 264.7 Macrophage Cells. Biol. Pharm. Bull..

[62] Alkanat M., Alkanat H.Ö (2024). D-Limonene Reduces Depression-like Behaviour and Enhances Learning and Memory through an Anti-Neuroinflammatory Mechanism in Male Rats Subjected to Chronic Restraint Stress. Eur. J. Neurosci..

[63] Lorigooini Z., Boroujeni S.N., Sayyadi-Shahraki M., Rahimi-Madiseh M., Bijad E., Amini-Khoei H. (2021). Limonene through Attenuation of Neuroinflammation and Nitrite Level Exerts Antidepressant-Like Effect on Mouse Model of Maternal Separation Stress. Behav. Neurol..

[64] Carvalho-Filho M.A., Ueno M., Hirabara S.M., Seabra A.B., Carvalheira J.B.C., De Oliveira M.G., Velloso L.A., Curi R., Saad M.J.A. (2005). S-Nitrosation of the Insulin Receptor, Insulin Receptor Substrate 1, and Protein Kinase B/Akt: A Novel Mechanism of Insulin Resistance. Diabetes.

[65] Lee C.H., Kim H.J., Lee Y.S., Kang G.M., Lim H.S., Lee S.H., Song D.K., Kwon O., Hwang I., Son M. (2018). Hypothalamic Macrophage Inducible Nitric Oxide Synthase Mediates Obesity-Associated Hypothalamic Inflammation. Cell Rep..

[66] Hewett S.J., Uliasz T.F., Vidwans A.S., Hewett J.A. (2000). Cyclooxygenase-2 Contributes ToN-Methyl-d-Aspartate-Mediated Neuronal Cell Death in Primary Cortical Cell Culture. J. Pharmacol. Exp. Ther..

[67] Chen Q., Luo Y., Kuang S., Yang Y., Tian X., Ma J., Mai S., Xue L., Yang J. (2017). Cyclooxygenase-2 Signalling Pathway in the Cortex Is Involved in the Pathophysiological Mechanisms in the Rat Model of Depression. Sci. Rep..

[68] Terzo S., Calvi P., Nuzzo D., Picone P., Allegra M., Mulè F., Amato A. (2023). Long-Term Ingestion of Sicilian Black Bee Chestnut Honey and/or D-Limonene Counteracts Brain Damage Induced by High Fat-Diet in Obese Mice. Int. J. Mol. Sci..

[69] Terzo S., Amato A., Calvi P., Giardina M., Nuzzo D., Picone P., Palumbo-Piccionello A., Amata S., Giardina I.C., Massaro A. (2025). Positive Impact of Indicaxanthin from Opuntia Ficus-Indica Fruit on High-Fat Diet–Induced Neuronal Damage and Gut Microbiota Dysbiosis. Neural Regen. Res..

[70] Restivo I., Basilicata M.G., Giardina I.C., Massaro A., Pepe G., Salviati E., Pecoraro C., Carbone D., Cascioferro S., Parrino B. (2023). A Combination of Polymethoxyflavones from Citrus Sinensis and Prenylflavonoids from Humulus Lupulus Counteracts IL-1β-Induced Differentiated Caco-2 Cells Dysfunction via a Modulation of NF-ΚB/Nrf2 Activation. Antioxidants.

[71] Schindelin J., Arganda-Carreras I., Frise E., Kaynig V., Longair M., Pietzsch T., Preibisch S., Rueden C., Saalfeld S., Schmid B. (2012). Fiji: An Open-Source Platform for Biological-Image Analysis. Nat. Methods.

