# Supplementary material for: Kumquat Fruit Administration Counteracts Dysmetabolism-Related Neurodegeneration and the Associated Brain Insulin Resistance in the High-Fat Diet-Fed Mice
Source: Int J Mol Sci. 2025 Mar 27;26(7):3077. doi: 10.3390/ijms26073077 (PMC11988715; doi:10.3390/ijms26073077)
Supplement: Supplementary file 1 [file ijms-26-03077-s001.zip › ijms-3515845-supplementary.pdf]

Table S1. Gene expression comparation between STD/HFD+K groups

**Symbol**	**Fold Regulation (comparing to STD group) Group HFD+K**
Apba3	0.55
Apbb1	0.44
Apoe	1.12
Bche	0.07
Clu	1.55
Gnb2	0.57
Gnb5	0.83
Gng10	0.08
Gng4	0.06
Gng5	0.09
Gng8	0.04
Ide	0.09
Igf2	0.07
Insr	0.54
Prkcd	0.10
